# Inter- and Intra-examiner Reliability of Quantitative Assessment of Respiratory Muscle Cross-Sectional Areas on Preoperative Chest CT Images: A Pilot Study

**DOI:** 10.7759/cureus.97690

**Published:** 2025-11-24

**Authors:** Kengo Shirado, Takashi Kido, Naoto Kawabata, Rina Nakao, Kenta Kawamitsu, Koji Mitsuzono, Manabu Yasuda

**Affiliations:** 1 Department of Rehabilitation, Iizuka Hospital, Iizuka, JPN; 2 Department of Central Radiology, Iizuka Hospital, Iizuka, JPN; 3 Department of Chest Surgery, Iizuka Hospital, Iizuka, JPN

**Keywords:** computed tomography, non-small cell lung cancer, reliability, respiratory muscles, respiratory sarcopenia

## Abstract

Introduction: Respiratory sarcopenia, defined as reduced respiratory muscle mass and strength, and has been reported as a potential prognostic factor in lung cancer. In patients undergoing non-small cell lung cancer (NSCLC) surgery, preoperative chest computed tomography (CT) is routinely performed and offers an opportunity to assess respiratory muscle volume. However, the reliability of CT-based measurements for different respiratory muscles has not been established, and standardized protocols are lacking. This study aimed to evaluate the inter-rater reliability of respiratory muscle volume measurements using preoperative CT images in patients with lung cancer.

Methods: Five patients who underwent lung cancer surgery at our hospital between July and August 2023 were selected. Four physical therapists with no prior experience in measuring muscle cross-sectional areas (CSAs) on chest CT images received a lecture on the measurement methods from a radiology technician. Respiratory muscle CSAs (pectoralis, intercostal, and latissimus dorsi muscles) were measured from preoperative chest CT images using an image analysis system. Measurements were performed four times on different days. Intra-class correlation coefficients (ICC) were used for intra- and inter-rater reliability assessments, applying a two-way mixed-effects model with absolute agreement: ICC (2, 1) for intra-rater and ICC (2, k) for inter-rater reliability. ICC ≥ 0.75 indicated excellent reliability.

Results: Participants had a median age of 75.0 (72.0, 79.0) years and body mass index of 23.0 (20.0-24.0). Four participants (80%) were at tumor stage I. Excellent intra-assessor reliability was demonstrated for all the respiratory muscles (pectoralis CSA [ICC: 0.908 to 0.996], intercostal CSA [ICC: 0.941 to 0.985], and latissimus CSA [ICC: 0.885 to 0.984]). Excellent inter-rater reliability was demonstrated for the pectoralis and intercostal CSAs.

Conclusions: This study confirmed excellent intra- and inter-rater reliability for measuring respiratory muscle CSAs using CT images in patients with preoperative lung cancer in the pectoral and intercostal muscles. This suggests that CT assessment of respiratory muscle volume may be widely used as a clinically useful indicator. Further standardization and simplification of this method will allow its use in broader clinical settings.

## Introduction

The European Working Group on Sarcopenia (EWGSOP) defined sarcopenia in 2010 [1] as a syndrome characterized by progressive and generalized loss of muscle mass and strength. Sarcopenia, characterized by reduced skeletal muscle mass and decreased muscle strength or physical function, requires assessment of skeletal muscle mass for a definitive diagnosis. In addition, decreased skeletal muscle mass in patients with non-small cell lung cancer (NSCLC) is a characteristic of cancer cachexia [2], and the preoperative and postoperative loss of skeletal muscle mass in patients with NSCLC has been reported to affect postoperative complications and prognosis [3-5]. Therefore, the preoperative assessment of skeletal muscle mass in patients with NSCLC is essential.

Respiratory sarcopenia has recently been proposed as a condition of decreased respiratory muscle strength and mass and has prognostic implications for respiratory diseases, particularly lung cancer and chronic obstructive pulmonary disease [6]. Although there are few reports on the impact of respiratory sarcopenia on postoperative lung cancer, it has been reported that respiratory muscle strength and chest muscle mass before lung cancer surgery affect survival [7]. A position paper on respiratory sarcopenia recommended computed tomography (CT) and ultrasonography to assess respiratory muscle mass [6]. In patients undergoing lung cancer surgery, chest CT is often performed as a preoperative assessment, and the respiratory muscle volume can be easily measured using CT images. However, currently, there are different target muscles for respiratory muscle cross-sectional areas (CSAs) assessment using CT, including the diaphragm [8,9] and intercostal [10], pectoralis [11-13], and vastus lateralis [14] muscles, and it has not been verified which respiratory muscle is the best for measurement or prognostic indicator.

There is a lack of knowledge regarding the reliability of respiratory muscle CSAs assessment and the need for standardization of measurement methods to improve the ability to assess respiratory muscle CSAs. Given the complexity of the scoring procedure, as the first step in respiratory muscle CSAs assessment, an analysis of the inter-rater reliability of respiratory muscle CSAs measurements is required. Therefore, this study aimed to analyze the inter-rater reliability of respiratory muscle volume assessment using preoperative CT in patients with lung cancer.

## Materials and methods

Study design, setting, and ethical considerations

This was an inter-rater reliability study of respiratory muscle CSA assessment using CT in patients who underwent lung cancer surgery. The study followed the guidelines for reporting reliability and agreement studies [15]. Informed consent was waived due to the retrospective design, and all CT data were de-identified prior to analysis. This study was approved by the ethics committee of Aso Iizuka Hospital (approval number: R-23115).

Population, sample, inclusion, and exclusion criteria

Participants were aged ≥18 years and were admitted for lung cancer surgery at our hospital between July and August 2023. Patients were excluded if they had stage 4 or higher lung cancer (PS: 4). Five patients were included in the study. This sample size was determined based on the recommendations of Walter et al. [16], assuming a null intra-class correlation coefficient (ICC) of 0.5, target ICC of 0.8, α = 0.05, power = 0.8, and a design involving four raters with four repeated measurements per subject.

Data collection

Four physiotherapists assessed muscle CSAs on chest CT images (SYNAPSE VINCENT, Fujifilm Corp., Tokyo, Japan). Each physical therapist had little experience in assessing muscle CSAs using CT but had received prior training from a radiologist with more than 10 years of experience. This was the only training received during data collection in this study.

Training in assessing muscle CSAs using CT took place in a face-to-face session lasting approximately one hour. Instructions were provided for image handling, anatomical structure identification (muscle, bone, and adipose tissue), tracing, and analysis. A manual was prepared for muscle evaluation, including the steps to be taken and points of interest. After the training program, the respiratory muscles of each participant were measured four times by four evaluators. Participants underwent a CT scan prior to lung cancer surgery. Lung and physical function parameters and surgical and oncological data were retrospectively collected after each respiratory muscle volume assessment.

Protocol for CT image evaluation

Preoperative chest CT examination was performed using a Revolution CT scanner (GE HealthCare Japan Corp., Tokyo, Japan). The CT examination was performed using a detector configuration of 128 rows×0.625 mm, tube voltage of 120 kVp, pitch of 0.992, and gantry rotation time of 0.35 s. The scale of the attenuation coefficients ranged from Hounsfield units (HU) -1024 to 3071. Patients were instructed to hold their breath at maximal inspiration in the raised arm position. The entire lung parenchyma, from the pulmonary apex to the diaphragm, was scanned in a craniocaudal direction. Reconstruction kernel: standard; matrix size: 512 × 512; field of view: adjusted to thoracic cavity. Respiratory muscle volume was assessed using SYNAPSE VINCENT. First, reconstructed images with a slice thickness of 5 mm and an interval of 5 mm were analyzed using CT histogram software ("X section" analysis tool, Advantage Window 4.4; GE Healthcare, Milwaukee, WI, USA). Second, the region of interest was placed at the outermost boundary of the muscle in a polygon. Third, CT histogram analysis was used to calculate the area of the muscles with HU ranging from -29 to 100, with reference to previous studies [14].

CT histogram analysis is a well-validated method for quantifying the area and mean attenuation of thigh muscles [17]. Finally, the mass of the respiratory muscles was calculated as the CSA.

Pectoralis CSA

**Figure 1:**
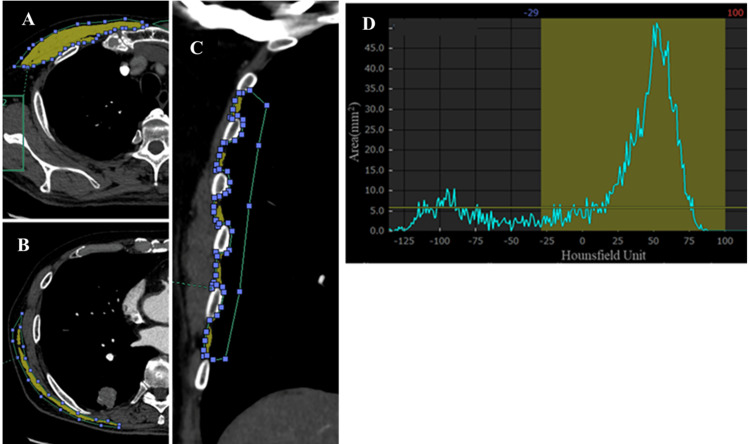
Method for measuring respiratory muscle cross-sectional areas (CSAs) (A) In the transverse section image, the region of interest (green line) is drawn at the outermost edge of the pectoralis muscles (pectoralis major and pectoralis minor) in the anterior thoracic region. (B) In the transverse section image, the region of interest (green line) is drawn at the outermost edge of the latissimus dorsi muscle at the level of the inferior border of the eighth thoracic vertebra. (C) In the coronal image, the region of interest (green line) is drawn at the outermost margin of the third to eighth intercostal muscles bilaterally. (D) CT histogram analysis automatically measured the CSAs and attenuation of each muscle (CT attenuation ranged from -29 to 100 HU). Each automatically measured muscle area is in the yellow range.

Pectoralis muscle measurements were performed as previously described [13]. The measurements were performed on a uniaxial slice obtained from a CT scan of the aortic arch. Transverse section images were visually identified on top of the aortic arch and scrolled toward the pulmonary apex to identify the first axial image over the arch. The bilateral pectoralis major and minor muscles were then identified in the anterior chest and summed to determine pectoral muscle volume (Figure 1A).

Latissimus dorsi CSA

Latissimus dorsi muscle measurements were performed as previously described [14]. A representative transverse section was selected at the inferior border of the eighth vertebral body because it was difficult to distinguish the border from the adjacent pectoralis major muscle at the level of the upper thoracic spine. The CSAs of the bilateral latissimus dorsi muscles were measured separately and both values were summed (Figure 1B).

Intercostal CSA

Intercostal muscle measurements were performed as previously described [14,17]. A representative midline coronal image was selected to visualize the lateral arcs of the first rib on both sides. All intercostal muscles were included in the midline coronal images and were largely unaffected by the rib angulation. The CSA of the third to eighth intercostal muscles on each side was measured, and the values for the left and right intercostal muscles were summed to determine the intercostal muscle volume (Figure 1C).

Statistical analysis

For descriptive analysis, categorical variables were expressed as numbers and percentages, and continuous variables were expressed as medians and interquartile ranges. To analyze inter- and intra-observer reliability, a two-way mixed-effects method with absolute agreement was considered, and ICC values were calculated [18]. Specifically, ICC (2, 1) was used for intra-rater reliability, representing a two-way mixed-effects model with absolute agreement for single measurements. For inter-rater reliability, ICC (2, k) was calculated using the average of four raters, as the same fixed raters evaluated all subjects. This approach is appropriate because the raters were not randomly selected, but rather fixed within the study design. For intra-rater reliability, the analysis was based on four measurements for each rater for each respiratory muscle: <0.4 was considered "poor," between 0.4 and 0.60 was considered "fair," between 0.60 and 0.75 was considered "good," and >0.75 was considered "excellent" [19]. The analysis was performed using EZR (Saitama Medical Center, Saitama Prefecture, Japan), a graphical user interface of R (The R Foundation for Statistical Computing, Vienna, Austria) [20].

## Results

There were five patients with a median age of 75.0 years [72.0, 79.0] and BMI of 23.0 [20.0, 24.0]. There were three males (60%). One patient (20%) had chronic obstructive pulmonary disease (COPD), four patients (80%) had stage I lung cancer, and one patient (20%) had stage II lung cancer (Table 1).

**Table 1:** Sociodemographic and clinical characteristics of participants n(%). Data are Median (Interquartile range). COPD, chronic obstructive pulmonary disease; BMI, body mass index; FEV 1%, percentage of predicted value for forced expiratory volume in 1 s; %VC, percentage of vital capacity.

Characteristics		Results
Age		75.0 [72.0, 79.0]
Sex	Male	3 (60%)
Site of lobectomy, n	Right middle	3 (60%)
	Right lower	1(20%)
	Left lower	1(20%)
Thoracoscopic, n		5 (100%)
COPD		1 (20%)
Preoperative chemotherapy		1 (20%)
BMI (kg/m^2^)		23.0 [20.0, 24.0]
FEV１%, %		71.4 [62.1, 72.8]
%VC, %		106.5 [104.5, 116.6]
Tumor stage, n	Ⅰ	4 (80%)
	Ⅱ	1 (20%)

The CSA of each respiratory muscle is shown in Table 2, and the results of the inter- and intra-examiner reliability analyses are shown in Table 3. Excellent intra-examiner reliability was demonstrated for all the respiratory muscles (intercostal [ICC: 0.941 to 0.985], pectoralis [ICC: 0.908 to 0.996], and latissimus dorsi [ICC: 0.885 to 0.984] muscles). Excellent inter-rater reliability was demonstrated for the intercostal (ICC: 0.914; 95% CI: 0.533 to 0.99) and pectoralis (ICC: 0.986; 95% CI: 0.945 to 0.98) muscles.

**Table 2:** Median and interquartile range of respiratory muscle cross-sectional areas (CSAs) per patient 95% CI, 95% confidence interval.

Patient	Muscles	Median	95% CI
A	Pectoralis	2406.839	2394.611, 2420.506
	Intercostal	320.866	302.791, 343.073
	Latissimus dorsi	1548.653	1531.136, 1666.753
B	Pectoralis	2972.074	2949.386, 3006.124
	Intercostal	479.989	451.659, 536.619
	Latissimus dorsi	1437.358	1401.140, 1684.719
C	Pectoralis	3177.675	3078.071, 3243.879
	Intercostal	559.728	545.816, 569.162
	Latissimus dorsi	2136.236	2046.826, 2239.074
D	Pectoralis	3735.267	3688.445, 3790.317
	Intercostal	540.376	494.209, 594.118
	Latissimus dorsi	2134.173	2023.974, 2239.900
E	Pectoralis	3248.733	3207.385, 3264.915
	Intercostal	628.519	592.216, 663.654
	Latissimus dorsi	1805.663	1784.904, 2009.853

**Table 3:** Intra-examiner and inter-examiner reliability of respiratory muscle cross-sectional areas (CSAs) according to muscles 95% CI, 95% confidence interval.

Muscles		Intra-Examiner Reliability			Inter-Examiner Reliability		
	Examiners	Results	95% CI	Correlation	Results	95% CI	Correlation
Pectoralis	Examiner 1	0.996	0.985, 1.000	Excellent	0.986	0.945, 0.998	Excellent
	Examiner 2	0.996	0.985, 1.000	Excellent			
	Examiner 3	0.908	0.651, 0.989	Excellent			
	Examiner 4	0.910	0.657, 0.990	Excellent			
Intercostal	Examiner 1	0.979	0.920, 0.998	Excellent	0.914	0.533, 0.990	Excellent
	Examiner 2	0.941	0.774, 0.993	Excellent			
	Examiner 3	0.961	0.850, 0.995	Excellent			
	Examiner 4	0.985	0.943, 0.998	Excellent			
Latissimus dorsi	Examiner 1	0.984	0.938, 0.998	Excellent	0.678	0.062, 0.956	Good
	Examiner 2	0.944	0.788, 0.994	Excellent			
	Examiner 3	0.885	0.562, 0.987	Excellent			
	Examiner 4	0.953	0.822, 0.995	Excellent			

## Discussion

This study aimed to analyze the intra- and inter-rater reliability of respiratory muscle CSA assessment using preoperative CT in patients with lung cancer. The main finding was that CT images showed excellent inter-rater reliability in measuring respiratory muscle CSAs in the intercostal and thoracic muscles. Several studies have reported that CT imaging is an objective method for assessing respiratory muscle mass [8-14]. However, the assessment of respiratory muscle CSAs using CT imaging is still uncommon, and only a few reports have validated its reliability. The high reproducibility of respiratory muscle CSA assessment supports the possibility that measurement methods using CT images can be reliably used to assess respiratory muscle CSAs in clinical practice. This study demonstrated inter-rater reliability for pectoralis CSA (ICC: 0.986), consistent with previous CT-based studies, which reported similar inter-rater ICC of 0.993 for pectoralis muscle area in patients with COPD [21], supporting the robustness of the measurement method.

Notably, the pectoralis CSA exhibited the highest intra- and inter-rater reliability among the muscles assessed. We assume that the pectoralis muscles are more straightforward for measuring CSAs than other muscles because the contours of the muscles are clearly visible on CT images. Many previous studies examining respiratory muscle CSAs have used the pectoralis muscle regardless of the disease, such as COPD and lung cancer [7,13,22,23], and this validation confirms the potential perception that measuring the area of the pectoralis muscle is easy. Second, the intercostal muscle CSAs showed the second-highest inter-rater reliability compared with the pectoralis muscle CSAs. The intercostal muscles consist of the innermost, internal, and external intercostal muscles [24]. Although it would be ideal to measure these three layers of the intercostal muscles separately, the spatial resolution of CT is limited; therefore, the CSAs of these muscles as a whole were measured based on previous studies [14]. We consider that the measurement of the intercostal CSAs, which consist of three layers, is easier and has the same high inter-rater reliability as the pectoral CSAs. On the other hand, the reason why the inter-rater reliability of the latissimus dorsal muscle area measurement was lower than that of other muscles may be due to the difficulty in identifying the measurement point and distinguishing it from the surrounding tissue because of its complex position and shape and its unclear border with other muscles. Thus, the fact that the structure and anatomical characteristics of the respiratory muscles affect the reliability of measurements may be an important finding for future improvements in the methods of assessing respiratory muscle CSAs.

In addition, respiratory muscle assessment using CT imaging is clinically important. Although ultrasound and CT imaging have been recommended for the assessment of respiratory muscle CSAs, one of the recently proposed diagnostic criteria for respiratory sarcopenia, no standardization of measurement methods has been established for any of the assessment methods [6]. Patients with respiratory diseases are often examined using chest CT imaging in routine medical care, and measurement of respiratory muscle CSAs using CT imaging can be a practical tool. Assessment of respiratory muscle CSAs based on histogram analysis using CT images, which was found to be highly reliable in this study, can reduce partial volume averaging effects and operator-dependent errors caused by manual region of interest drawing. This method has the potential to provide an objective and accurate measure for assessing a patient's respiratory muscles. In addition, as the assessment can focus on specific muscle groups, it is expected that changes in respiratory function will be detected according to the type and progression of the disease and will contribute to the formulation of more appropriate rehabilitation strategies.

This study had several limitations. First, the CT equipment and analysis software used for the measurements were limited. Further studies are required to determine whether similar reliability can be achieved using other equipment and software. Second, although the sample size was determined based on established statistical recommendations for reliability studies, the small number of participants (n=5) limits generalizability and may lead to wide confidence intervals in some measurements. Third, the study population was specific, and further studies with broader and more diverse samples are needed. Fourth, improvements in the standardization of analytical procedures and measurement points, especially for the latissimus dorsi, remain necessary. Fifth, the raters were newly trained and not blinded across sessions, which may have introduced a learning effect. These limitations underscore the need for further multi-center validation studies with larger and more diverse samples.

## Conclusions

This study confirmed the excellent intra- and inter-rater reliability of measuring respiratory muscle CSAs using CT images in patients with preoperative lung cancer in the pectoral and intercostal muscles. This suggests that CT assessment of respiratory muscle volume may be widely used as a clinically useful indicator. Further standardization and simplification of this method will allow its use in more centers.
